# Editorial: On the destabilization of maladaptive memory: updates and future perspectives

**DOI:** 10.3389/fnbeh.2023.1351704

**Published:** 2024-01-05

**Authors:** Andressa Radiske, Emma N. Cahill, Amy L. Milton, Martín Cammarota

**Affiliations:** ^1^Memory Research Laboratory, Brain Institute, Federal University of Rio Grande do Norte, Natal, Brazil; ^2^Edmond and Lily Safra International Institute of Neuroscience, Macaiba, Brazil; ^3^School of Physiology, Pharmacology and Neuroscience, University of Bristol, Bristol, United Kingdom; ^4^Department of Psychology, University of Cambridge, Downing Site, Cambridge, United Kingdom

**Keywords:** reconsolidation, restabilization, addiction, fear and anxiety, neuropharmacology

Intense fear induces persistent memories that can result in exacerbated maladaptive avoidance and lead to the development of phobias and other anxiety disorders. Similarly, drug addiction has been related to the formation of strong pervasive memories that trigger craving and relapse. Long-lasting consolidated memories can become destabilized when reactivated and, to endure, must undergo a protein synthesis-dependent restabilization in a process called reconsolidation (Nader et al., 2000). Because inhibition of memory restabilization appears to cause amnesia in laboratory animals, it has been suggested that therapies based on the pharmacological or behavioral modulation of this process may be useful to treat anxiety or addiction-related disorders (Monfils and Holmes, 2018). Although our knowledge about the molecular basis of memory reconsolidation has grown exponentially during the last two decades, there is no widespread reconsolidation therapy yet, and large-scale clinical trials are yet to be conducted. Furthermore, it is usually impractical and sometimes unethical, to reenact the behavioral and emotional conditions required for successful memory destabilization during psychotherapy, and the boundary conditions for ‘real life' memories (as opposed to those generated by experimental procedures) are not fully understood. To overcome this limitation, it has been argued that basic research should focus on elucidating the molecular and physiological signatures of reactivation-induced memory destabilization to determine specific reconsolidation biomarkers (Radiske et al., 2020; Milton et al., 2023). In this Research Topic, we have compiled original research articles presenting the latest findings on the mechanisms underlying memory destabilization.

Exploring the boundary conditions of memory reconsolidation, Cheng et al. contribute to this Research Topic showing that retrieval-induced memory destabilization of a well-learned instrumental memory is influenced by the delay between the end of training and the reactivation session. The authors found in rats that injection of the non-competitive NMDA receptor antagonist MK-801 before a reactivation session carried out 48 h, but not 24 h, after training impairs instrumental performance at test. Interestingly, the amnesia induced by MK-801 was not observed when different behavioral conditions were introduced between training and the reactivation session. These approaches included the interposition of a context extinction session, an additional reactivation session, or even the simple experience of being handled and injected with a vehicle solution. This work highlights the scarce data on reactivation-induced memory destabilization of instrumental memories and the need for additional research on this topic.

Drug-related cues elicit craving and relapse even after long periods of abstinence. It has been proposed that disrupting reconsolidation-related plasticity may be an effective strategy in preventing recurrence of substance abuse (Milton and Everitt, 2010; Torregrossa and Taylor, 2013). However, one of the main limitations of this approach is the lack of a pharmacological target appropriate for use in humans. Shi et al., describe the role of the mTORC1 and its downstream effectors in the maintenance of a cocaine contextual memory in mice. Using the conditioned place preference task, the authors showed that phosphorylation of the mTORC1 target P70S6K, but not of the eIF4E–eIF4G complex, in the nucleus accumbens and the hippocampus is enhanced following memory reactivation. They also showed that inhibition of mTORC1 and P70S6K after reactivation abolishes cocaine-related contextual memory restabilization. Given that rapamycin, a potent inhibitor of mTORC1, is FDA approved for human use and safely administered in short-term treatments, the discoveries of Shi and coworkers suggest that mTOR could be a key pharmacological target for disrupting the reconsolidation of drug memories.

Prediction error serves as a boundary condition for the reconsolidation of various memory types, but it is still unclear how different characteristics of perceived mismatches affect the updating of habit memories (Vousden and Milton, 2017; Piva et al., 2020). Tavares et al. contribute to this topic by exploring temporal parameters of prediction errors in the reconsolidation of an appetitive operant conditioning memory. The authors studied the role of the basolateral amygdala in updating long-term memories after an unexpected delay in the time of reward availability (temporal prediction error). They found that modifying the interval preceding reward availability during the error session changed the temporal expectation of the reward in a test session conducted 24 h later. Based on these observations, Tavares et al. concluded that the variation of reward contingency during instrumental memory reactivation could be a successful trigger for memory destabilization, carrying enough unpredictability to induce instrumental memory destabilization.

In the last work of this Research Topic, Noël summarizes current knowledge supporting the combination of non-invasive brain stimulation techniques and memory updating to treat individuals with substance-related disorders. He also acknowledges the preliminary nature of the available evidence in this field and discusses the necessity for further research to identify optimal conditions for reducing compulsive behaviors through the reconsolidation process.

In conclusion, this Research Topic emphasizes recent discoveries from basic, translational, and clinical research studies concerning the destabilization of maladaptive memories. This article Research Topic provides new insights into the conditions that facilitate reactivation-induced memory destabilization, as well as the novel molecular mechanisms and signaling pathways associated with memory destabilization. Additionally, it explores non-invasive interventions aimed at inducing maladaptive memory destabilization. These insights not only enrich the theoretical framework of memory destabilization but also provide substantial contributions for future research on the modulation of maladaptive memories.

## Author contributions

AR: Writing—original draft, Writing—review & editing. EC: Writing—original draft, Writing—review & editing. AM: Writing—original draft, Writing—review & editing. MC: Writing—original draft, Writing—review & editing.
